# Soluble α-Klotho levels, glycemic control and renal function in US adults with type 2 diabetes

**DOI:** 10.1007/s00592-022-01865-4

**Published:** 2022-03-14

**Authors:** Stefano Ciardullo, Gianluca Perseghin

**Affiliations:** 1 Department of Medicine and Rehabilitation, Policlinico di Monza, Via Modigliani 10, 20900 Monza, MB Italy; 2 Department of Medicine and Surgery, Università degli Studi di Milano Bicocca, Milan, Italy

**Keywords:** Klotho, CKD, Diabetes, HbA1c

## Abstract

**Aims:**

Soluble Klotho (s-Klotho) is associated with chronic kidney disease (CKD) and aging, but little is known on its relationship with chronic micro- and macro-vascular complications of type 2 diabetes and glycemic control. Here, we evaluate the association between s-Klotho levels, glycemic control and renal function in patients with type 2 diabetes (T2D).

**Methods:**

This is a cross-sectional study including 2989 patients with T2D and available s-Klotho measurements from the 2007–2016 cycles of the National Health and Nutrition Examination Survey (mean ± SE, age: 60.0 ± 0.2 years, BMI 33.3 ± 0.2 kg/m^2^, 46.7 ± 1.3% female). Determination of s-Klotho concentrations was performed with a sandwich ELISA test.

**Results:**

Patients with higher s-Klotho levels were younger, more frequently female and had a lower prevalence of CKD and higher HbA1c levels. In multivariable linear regression models adjusting for age, race-ethnicity and BMI, both estimated glomerular filtration rate (B = 2.21, 95% CI 1.41–3.01, *p* < 0.001) and hemoglobin A1c (B = 37.38, 95% CI 28.91–45.86, *p* < 0.001) were positively associated with s-Klotho, while no significant association was found with cardiovascular disease. Results were confirmed when analyses were performed in men and women separately. No significant differences were identified between patients with an albuminuric or non-albuminuric CKD phenotype.

**Conclusions:**

s-Klotho levels are dependent on kidney function and glycemic control in patients with T2D. Additional studies elucidating the mechanisms linking glycemic control and s-Klotho levels and exploring their predictive ability of clinically meaningful outcomes in patients with diabetes are needed.

**Supplementary Information:**

The online version contains supplementary material available at 10.1007/s00592-022-01865-4.

## Introduction

α-Klotho (referred here as Klotho) was serendipitously discovered in 1997 as a gene linked to aging in mice, with reduced gene expression being linked to a shortened life span [1]. It was later discovered that the gene codes for a transmembrane protein functioning as the obligate co-receptor for fibroblast growth factor 23 (FGF-23), a phosphaturic hormone essential for maintaining mineral homeostasis by regulating phosphorus, parathyroid hormone and vitamin D concentrations [2, 3]. Its expression is the highest in the distal tubules of the kidneys [4] and because its ectodomain seems to be shed constitutively, circulating levels of Klotho (referred here as soluble Klotho, s-Klotho) are thought to be a surrogate marker of renal Klotho expression and probably of functional nephron number [5]. In agreement with this hypothesis, it has been shown that s-Klotho levels decrease proportionately with decreasing estimated glomerular filtration rate (eGFR) in both cross-sectional and longitudinal studies performed in patients with chronic kidney disease (CKD) [6–9].

Klotho deficiency has also been shown to be associated with endothelial dysfunction and vascular aging [10], with several reports highlighting lower circulating levels in patients with several chronic conditions including hypertension, cardiovascular disease (CVD) and Alzheimer disease [11].

Few studies to date investigated s-Klotho levels in patients with diabetes and little information is available on its association with chronic micro- and macro-vascular complications as well as with glycemic control [12, 13].

Since in the 2007–2016 cycles of the National Health and Nutrition Examination Survey (NHANES) serum s-Klotho was measured for the first time in a large, US nationally representative sample, data from the survey were used in the present study to identify determinants of its concentration in US adults with type 2 diabetes (T2D).

## Materials and methods

This is an analysis of data obtained in the 2007–2008 to 2015–2016 cycles of NHANES, a series of cross-sectional surveys conducted in the United States by the National Center for Health Statistics of the Centers for Disease Control and Prevention. Participants are selected from the US non-institutionalized, civilian population of all ages using a stratified, multistage, clustered probability sampling design. In order to derive reliable estimates with sufficient statistical power, oversampling of non-Hispanic black, Hispanic and Asian persons, people with low income and older adults is performed. The survey consists of a structured interview conducted in the home, followed by a standardized health examination performed at a mobile examination center, which includes a physical examination as well as laboratory tests. Full methodology of data collection is available elsewhere [14]. Among persons selected for the NHANES from 2007 to 2016, the response rates ranged from 75 to 55%. The original survey was approved by the Centers for Disease Control and Prevention Research Ethics Review Board and written informed consent was obtained from all adult participants. The present analysis was deemed exempt by the Institutional Review Board at our institution, as the dataset used in the analysis was completely de-identified.

### Laboratory tests and clinical data

Participants reported age, sex, race and ethnic group (non-Hispanic white, non-Hispanic black, Hispanic, or other), a previous diagnosis of diabetes (and age at diagnosis) as well as a previous history of CVD (defined as a composite of coronary artery disease and stroke/transient ischemic attacks). Body measurements including height (cm), weight (kg) and waist circumference (cm) were ascertained during the mobile examination center visit; we calculated body mass index (BMI) as weight in kilograms divided by the square of the height in meters. After the participant rested in a seated position for 5 min, blood pressure was measured three times on the same arm with a mercury sphygmomanometer by trained medical personnel. The average of the three measures was used as the representative value in this analysis. Hypertension was defined as a systolic blood pressure (SBP) value ≥ 140 mmHg and/or a diastolic blood pressure (DBP) value ≥ 90 mmHg or currently taking antihypertensive drugs [15]. Diabetes was defined in accordance with the American Diabetes Association criteria if any of the following conditions were met: 1) A self-reported diagnosis of diabetes. 2) Use of anti-diabetic drugs. 3) A Hemoglobin A1c (HbA1c) level ≥ 6.5% (48 mmol/mol). 4) A fasting plasma glucose ≥ 126 mg/dl. 5) A random plasma glucose ≥ 200 mg/dl [16]. We excluded patients with probable type 1 diabetes (defined as an age of onset < 30 years and currently taking insulin as the only anti-diabetic drug).

HbA1c was measured with the use of high-performance liquid chromatography methods. Laboratory methods used to measure glucose, creatinine, calcium, phosphorus, albumin and other metabolites are reported in detail elsewhere [17]. Estimated glomerular filtration rate (eGFR) was computed according to the Chronic Kidney Disease Epidemiology Collaboration (CKD-EPI) equation. Based on the measured urine albumin to creatinine ratio (UACR), participants were defined as having normo-albuminuria (UACR < 30 mg/g), micro-albuminuria (UACR between 30 and 300 mg/g) or macro-albuminuria (UACR ≥ 300 mg/dL).

All respondents were asked if they had taken any prescription medications in the past 30 days. If so, they were asked to show the interviewers the containers; when they were not available, participants reported the medication names. All drugs were converted into standard generic drug name, and we categorized them into specific classes of anti-diabetic drugs.

### s-Klotho measurements

Determination of s-Klotho concentrations was performed on frozen samples stored at − 80 °C that were received and tested during the period 2019–2020. Available pristine serum samples from 40–79 years old participants, who gave consent for their samples to be used in future research, were analyzed with a sandwich ELISA test (IBL-International, Japan) at the Northwest Lipid Metabolism and Diabetes Research Laboratories, University of Washington. All sample analyses were performed in duplicate, and the average of the two values was used to calculate the final value, according to the manufacturer’s protocol; the results were checked to meet the laboratory’s standardized criteria for acceptability. The assay is reported to have a sensitivity of 6 pg/ml [18]; it was used in several prior publications and showed reasonable correlation with more labor-intensive assay methods using synthetic antibodies and immune-precipitation-immunoblots. Additional information is available at the NHANES website.

### Study sample

A total of 16,862 participants aged 40–79 years attended a mobile examination center visit. We initially excluded individuals without diabetes or with probable type 1 diabetes (defined as a diagnosis at age < 30 years and the use of insulin as the only anti-diabetic therapy), leading to a population of 3659 patients with T2D. Among these, 670 did not have a s-Klotho measurement, leading to a final sample of 2989 patients with T2D and S-Klotho data (Supplementary Fig. 1).

### Statistical analysis

All analyses were conducted using Stata version 16.0 (StataCorp, College Station, TX), accounting for the complex survey design of NHANES. We used appropriate weighting for each analysis, as suggested by the NCHS. Sample weights are used to account for oversampling of certain populations and survey nonresponse.

We first examined participants’ characteristics by median s-Klotho levels. These were summarized as numbers and weighted proportions for categorical variables and as weighted means ± Standard Error (SE) for continuous variables. For eGFR, we also reported the proportion of participants in each group below or above clinically relevant cut-points. Features were compared using linear regression for continuous variables and the design-adjusted Rao-Scott Chi-square test for categorical variables.

Multivariable linear regression analysis was performed in order to evaluate the effect of different variables on circulating s-Klotho levels. The contribution of glycemic control and CVD complications was evaluated after adjustment for age, sex and race ethnicity. Sensitivity analyses were performed by repeating the analysis in men and women separately. A two-tailed value of p < 0.05 was considered statistically significant.

## Results

Clinical and biochemical features of the study population according to s-Klotho levels are shown in Table 1. Mean (SE) age was 60.0 (0.2) years, with 46.7% women. The median s-Klotho level was 805.4 pg/ml (25^th^–75^th^ percentile = 646–1020 pg/ml). Mean eGFR was 83.6 ml/min/1.73m^2^, with 16.4% of patients having an eGFR < 60 ml/min/1.73m^2^ and 25.3% having an UACR > 30 mg/g.

**Table 1:** Features of the study population according to s-Klotho levels

	Total (*n* = 2989)	Below median (*n* = 1496)	Above median (*n* = 1493)	*p* value
Age (years)	60.0 ± 0.2	61.1 ± 0.4	58.9 ± 0.3	< 0.001
Age group (%)				0.001
40–49	17.3 ± 1.0	14.6 ± 1.3	20.1 ± 1.4	
50–59	29.4 ± 1.2	27.8 ± 1.8	31.1 ± 1.8	
60–69	32.5 ± 1.4	32.9 ± 1.9	31.9 ± 2.0	
70–79	20.8 ± 0.7	24.6 ± 1.2	16.8 ± 1.1	
Female sex (%)	46.7 ± 1.3	44.1 ± 1.8	49.4 ± 1.8	0.046
Race-ethnicity (%)				0.027
Non-Hispanic white	60.2 ± 2.3	57.4 ± 2.8	62.9 ± 2.4	
Hispanic	16.7 ± 1.7	19.1 ± 2.0	14.6 ± 1.7	
Non-Hispanic black	14.0 ± 1.2	14.6 ± 1.5	13.5 ± 1.2	
Other	9.0 ± 0.9	9.0 ± 1.1	9.1 ± 1.1	
BMI (kg/m^2^)	33.3 ± 0.2	33.3 ± 0.2	33.3 ± 0.4	0.714
T2D duration (years)	8.5 ± 0.2	9.4 ± 0.3	7.6 ± 0.3	< 0.001
*Laboratory parameters*				
HbA1c (%)	7.4 ± 0.0	7.1 ± 0.1	7.6 ± 0.1	< 0.001
eGFR (ml/min/1.73m^2^)	83.6 ± 0.5	79.8 ± 0.7	87.6 ± 0.7	< 0.001
UACR (mg/g)	113.4 ± 13.2	145.8 ± 22.5	79.5 ± 9.9	0.051
Calcium (mg/dL)	9.4 ± 0.0	9.5 ± 0.0	9.4 ± 0.0	0.626
Phosphorus (mg/dL)	3.7 ± 0.0	3.7 ± 0.0	3.8 ± 0.0	0.731
*Comorbidities*				
Hypertension (%)	71.3 ± 1.2	74.5 ± 1.6	67.9 ± 1.7	0.034
eGFR category (%)				< 0.001
> 60	83.6 ± 0.7	78.0 ± 1.2	89.6 ± 1.0	
45–60	10.2 ± 0.8	13.3 ± 1.3	7.0 ± 0.9	
30–45	4.0 ± 0.5	5.4 ± 0.7	2.5 ± 0.4	
< 30	2.1 ± 0.3	3.2 ± 0.6	0.9 ± 0.3	
Cardiovascular disease	21.5 ± 1.0	23.2 ± 1.6	19.7 ± 1.5	0.125
UACR (%)				0.108
< 30	74.7 ± 1.1	73.3 ± 1.7	76.1 ± 1.4	
30–300	19.2 ± 1.1	19.5 ± 1.5	18.9 ± 1.3	
> 300	6.1 ± 0.7	7.2 ± 1.0	5.0 ± 0.7	
*Glucose-lowering drugs*				
Metformin (%)	52.0 ± 1.1	54.2 ± 1.7	49.6 ± 1.8	0.091
Insulin (%)	18.6 ± 1.0	21.0 ± 1.5	16.0 ± 1.3	0.010
Sulfonylureas (%)	26.8 ± 1.1	27.9 ± 1.6	25.7 ± 1.4	0.256
GLP1-RA (%)	2.9 ± 0.5	2.4 ± 0.6	3.4 ± 0.6	0.291
SGLT2-i (%)	0.5 ± 0.2	0.6 ± 0.4	0.3 ± 0.1	0.448
DPP4-i (%)	8.0 ± 0.7	8.4 ± 1.1	7.6 ± 1.0	0.620
TZD (%)	8.5 ± 0.7	8.9 ± 1.2	8.1 ± 1.0	0.651

Data are expressed as weighted proportions (± Standard Error (SE)) for categorical variables and as weighted means ± SE for continuous variables. Linear regression and Rao-Scott Chi-square test were used to compare groups. The median s-Klotho level was 805.4 pg/ml

*BMI* Body mass index, *UACR* Urinary albumin creatinine ratio, *HbA1c* Hemoglobin A1c, *eGFR* Estimated glomerular filtration rate

Patients with s-Klotho levels below the median were significantly older (61.1 vs 58.9 years, *p* < 0.001) and less frequently female (44.1 vs 49.4%, *p* = 0.046), while no significant difference was found in BMI. Among laboratory parameters, HbA1c levels were significantly lower in patients with lower s-Klotho levels (7.1 vs 7.6%, *p* < 0.001), together with a lower eGFR (79.8 vs 87.6 ml/min/1.73m^2^, *p* < 0.001), while a non-significant trend was identified in terms of UACR. No differences were identified in phosphorus and calcium concentrations.

Moreover, patients with reduced s-Klotho concentrations also displayed a higher prevalence of hypertension and CKD, while no statistically significant differences were identified in both CVD and micro- and macro-albuminuria. Finally, no significant differences were identified in glucose-lowering drugs, with the exception of insulin, which was more commonly used in patients with lower s-Klotho levels (21.0% vs 16.0%, *p* = 0.010). Nonetheless, mean s-klotho levels in patients treated with insulin did not significantly differ from those of patients treated with metformin or sulfonylureas (818.5 vs 824.4, *p* = 0.763).

### Impact of kidney function and glycemic control

**Fig. 1:**
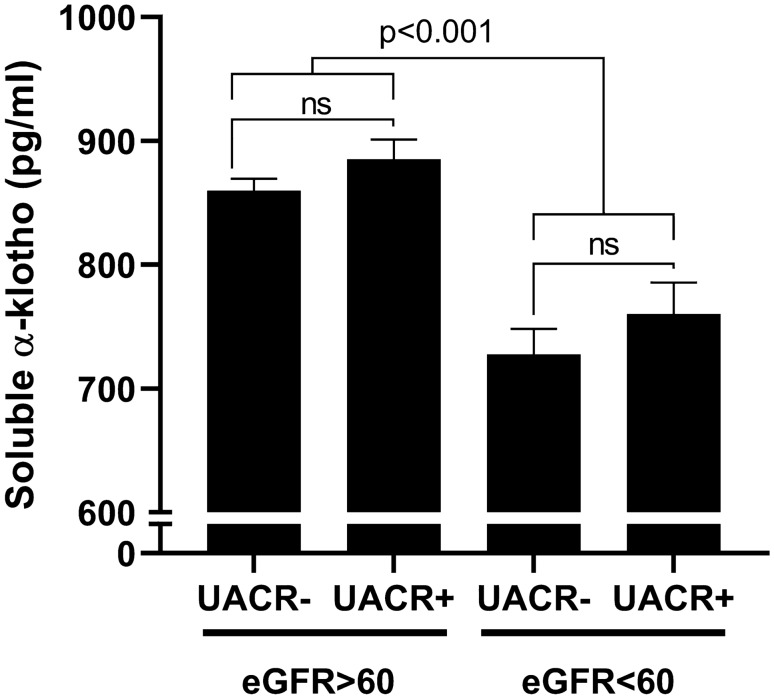
s-Klotho levels according to kidney function (estimated glomerular filtration rate, eGFR) and albuminuria

Figure 1 shows the relative impact of reduced eGFR and increased UACR on s-Klotho concentration. We did not identify a significant difference in s-Klotho levels in both patients with and without reduced eGFR when stratified according to UACR values. On the other hand, compared with patients without reduced eGFR, both patients with an albuminuric and non-albuminuric CKD phenotype showed significantly decreased s-Klotho levels (*p* < 0.001).

**Fig. 2:**
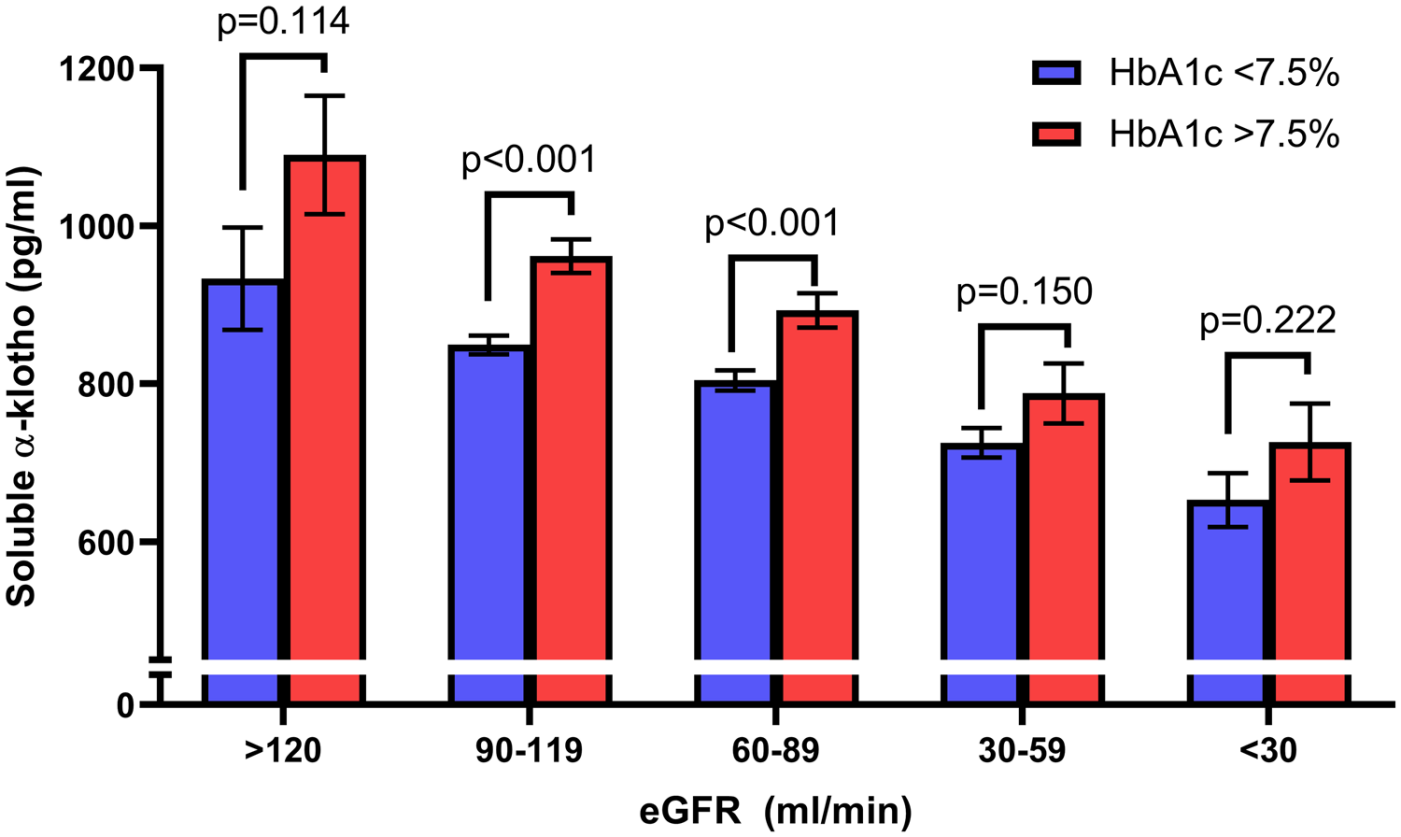
s-Klotho levels according to kidney function and glycemic control

To evaluate the contribution of glycemic control on s-Klotho concentration, we stratified the study population based on eGFR and Hba1c levels using a cut-off point of 7.5% (which was chosen as close to the median HbA1c value in the entire sample). As shown in Fig. 2, patients with worse glycemic control displayed higher s-Klotho concentrations, particularly if renal function was preserved. Sensitivity analyses performed using different HbA1c cut-offs and performed separately in men and women confirmed these results (Supplementary Table 1).

The direct relationship between s-klotho and eGFR was also evident when NHANES participants without T2D (*n* = 10,776) were evaluated (Supplementary Table 2).

### Independent predictors of s-Klotho concentrations

To evaluate the independent contribution of glycemic control, kidney function and cardiovascular disease on s-klotho concentration, we performed a multivariable linear regression analysis adjusting for age, sex, BMI and race ethnicity. As shown in Table 2, a positive and statistically significant association was found between s-Klotho and increasing eGFR and HbA1c levels (*p* < 0.001 for both). A non-significant trend was identified suggesting lower levels for women compared with men (*p* = 0.057). A negative relationship was also identified between s-klotho and UACR, particularly in women. On the other hand, we did not identify any significant relationship between s-Klotho and age, BMI and CVD.

**Table 2:** Multivariable linear regression model assessing the contribution of several predictors on s-Klotho levels in the studied population

	Both sexes			Men			Women		
Variables	B	95% CI	*p* value	B	95% CI	*p* value	B	95% CI	*p* value
Age (years)	0.075	– 1.684–1.835	0.932	– 0.032	– 2.305–2.241	0.978	0.213	– 2.583–3.009	0.880
Race– ethnicity	6.532	– 7.260–20.324	0.349	– 2.455	– 22.232–17.323	0.806	13.179	– 5.538—31.896	0.165
BMI (kg/m^2^)	0.197	– 2.109–2.503	0.866	– 2.988	– 5.902 to – 0.075	0.045	2.175	– 1.040–5.390	0.182
UACR (mg/g)	– 0.017	– 0.033 – 0.000	0.044	– 0.013	– 0.040–0.013	0.314	– 0.018	– 0.034 to – 0.001	0.036
eGFR (ml/min/1.73m^2^)	2.211	1.405–3.017	< 0.001	2.459	1.356–3.562	< 0.001	2.140	1.069–3.210	< 0.001
CVD	– 13.086	– 46.579–20.407	0.439	0.557	– 41.608–42.721	0.979	– 18.996	– 69.597–31.605	0.457
HbA1c (%)	37.381	28.907–45.856	< 0.001	41.780	28.934–54.626	< 0.001	32.678	19.762–45.595	< 0.001

*BMI* Body mass index, *CVD* Cardiovascular disease, *eGFR* Estimated glomerular filtration rate, *HbA1c* Hemoglobin A1c, *UACR* Urine albumin to creatinine ratio, *CI* Confidence interval

## Discussion

This is to our knowledge the first study evaluating determinants of s-Klotho concentrations in a large, unselected sample of patients with T2D from the general population. Our main findings are that s-Klotho levels are significantly higher in patients with preserved kidney function and worse glycemic control. Both relationships were robust in both men and women and after adjustment for potential confounders.

Several smaller studies investigated the relationship between s-Klotho levels and eGFR, demonstrating lower circulating levels being associated with worsening kidney function, particularly in patients on hemodialysis. Our study expands on these results by focusing on the largest population to date of patients with T2D with and without CKD and by demonstrating that reduced eGFR, and not albuminuria is a significant determinant. This was evident by the lack of difference in s-Klotho concentrations between patients with albuminuric and non-albuminuric CKD phenotypes. While both phenotypes are associated with increased mortality rates in patients with T2D [19], we may speculate that s-Klotho concentrations might not capture glomerular disease leading to albumin excretion, but rather be a marker of the number of functional nephrons and therefore be more strictly related to eGFR. Additional studies are needed to evaluate whether low s-Klotho may be considered also a risk factor, and not only a marker, of renal impairment, as recently suggested by a longitudinal study performed on older adults [6].

The second association, related to higher s-Klotho levels being present in patients with worse glycemic control, independently of kidney function, is a novel finding. The association was robust in both men and women, across groups of eGFR and after adjustment for age, sex, race ethnicity and CVD. Since Klotho is reported as an anti-aging gene and T2D has been considered by some as a condition of accelerated senescence, one would expect lower levels in patients with worse diabetes control. This unexpected finding might be related to the effects of glycosuria increasing metabolic demands in the proximal and distal kidney tubules, which in turn might lead to increased klotho expression and/or cleavage resulting in higher serum concentrations. It should be noted that a previous study showed that exposure to high glucose levels did not alter Klotho expression in human isolated tubular epithelial cells [12]. It is nonetheless possible that a different response might be elicited in the intact nephron, based on potential paracrine and endocrine regulators.

The lack of association between s-Klotho and CVD is in line with a previous study by Zhang et al. in patients with T2D [20]. The authors did not identify significant relationships between micro- and macro-vascular diabetes complications and s-Klotho concentrations, with the exception of diabetic nephropathy.

Our study has several strengths. It is the largest study to date performed in an unselected sample of US adults with T2D, including both sexes and patients of different ethnic background. The high number of patients included yielded high statistical power to perform subgroup and sensitivity analyses and evaluate the impact of several predictors in multivariable models. Being based on NHANES data, our results have a high degree of external validity as the purpose of the survey is to be representative of the overall US population. Finally, acquisition of clinical, laboratory and anthropometric data was standardized and homogenous.

The present study also has some limitations that need to be acknowledged. First, its cross-sectional design does not allow causal inference to be demonstrated. On this aspect, future longitudinal investigations might shed light on the potential ability of s-Klotho to predict clinically relevant outcomes in patients with T2D. In fact, given that s-klotho levels have been shown to associate with several variables, it is difficult to confer a clear biological meaning to our observation.

Second, concern has been raised as to the accuracy of the ELISA kit commercially available for s-Klotho measurement [21, 22]. Although the assay used in the present study might be less specific compared with highly labor-intensive immunoprecipitant immune blot assay methods [23], our results confirm previous findings on the association between s-Klotho and CKD. Moreover, it could be hypothesized that inaccuracy in the assay might lead to bias results toward the null, rather than producing a positive finding. Third, assessment of kidney function was performed by the CKD-EPI equation and not by more accurate techniques such as creatinine clearance or the gold standard inulin clearance; nevertheless, equations based on serum creatinine are well-suited for large epidemiological studies. Fourth, as data for the current study were obtained before 2015, the proportion of patients treated with drugs now representing the worldwide first choice in treating T2D (i.e., glucagon-like peptide-1 receptor agonists and sodium-glucose co-transporter 2 inhibitors) is very limited. Given that these agents might impact s-klotho levels, results obtained in the current study might not be applied to patients treated with these novel agents.

## Conclusions

In conclusion, in this large population-based cross-sectional study including 2989 patients with T2D, serum levels of s-Klotho are positively associated with eGFR and HbA1c levels. On the other hand, no significant role was identified for obesity and CVD. Additional studies elucidating the mechanisms explaining the effect of worse glycemic control on s-Klotho concentration and exploring its predictive ability of clinically meaningful events are needed.

## Supplementary Information

Below is the link to the electronic supplementary material.

Supplementary file1 (DOCX 68 kb)

## Data Availability

All data analyzed during this study are publicly available at the NHANES website.

## Declarations

### Code availability

Code is available from the corresponding author upon reasonable request.
