# Supplementary material for: Soluble α-Klotho levels, glycemic control and renal function in US adults with type 2 diabetes
Source: Acta Diabetol. 2022 Mar 14;59(6):803–9. doi: 10.1007/s00592-022-01865-4 (PMC9085659; doi:10.1007/s00592-022-01865-4)
Supplement: Supplementary file 1 — Supplementary file1 (DOCX 68 kb) [file 592_2022_1865_MOESM1_ESM.docx]

**ONLINE SUPPLEMENT**

**Soluble α-Klotho levels, glycemic control and renal function in US adults with type 2 diabetes**

**Running Title**: Advanced fibrosis in type 2 diabetes

**Authors:** Stefano Ciardullo^1,2^, Gianluca Perseghin^1,2^

**Affiliations**: ^1^Department of Medicine and Rehabilitation, Policlinico di Monza, Monza, Italy ^2^Department of Medicine and Surgery, University of Milano Bicocca, Milan, Italy

**Correspondence**: Prof Gianluca Perseghin, Department of Medicine and Surgery, Università degli Studi di Milano Bicocca & Depart­ment of Medicine and Rehabilitation, Policlinico di Monza, Via Modigliani 10, 20900 Monza (MB), Italy, Email: gianluca.perseghin@policlinicodimonza.it or gianluca.perseghin@unimib.it

Phone +39 039 2810430

**Supplementary Figure 1** Flow-chart of the study participants.


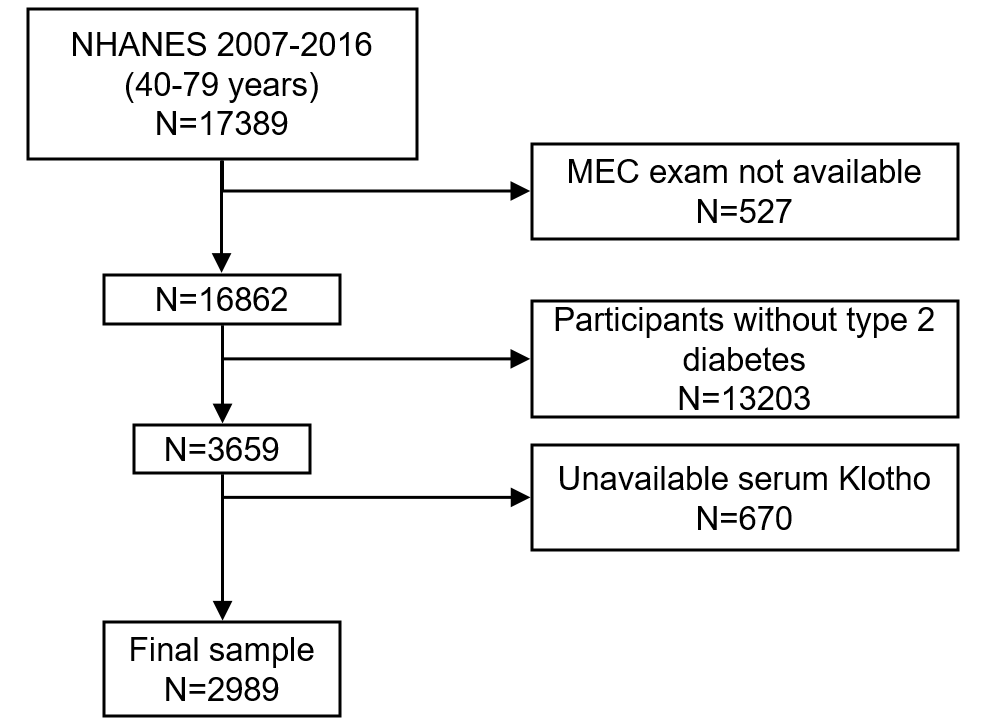


Abbreviations: NHANES, National Health and Nutrition Examination Survey; MEC, mobile examination center.

**Supplementary Table 1** Mean s-Klotho levels according to renal function and glycemic control in men and women with type 2 diabetes.

|  | **Men** | | | **Women** | | |
| --- | --- | --- | --- | --- | --- | --- |
| **eGFR (ml/min/1.73m^2^)** | **HbA1c<7.5** | **HbA1c≥7.5** | **Total** | **HbA1c<7.5** | **HbA1c≥7.5** | **Total** |
| >120 | 900.7 (114.3) | 1,066.3 (114.9) | 1,002.9 (84.5) | 943.7 (69.0) | 1,099.6 (89.1) | 1,032.3 (58.7) |
| 90-119 | 830.8 (18.0) | 975.8 (23.6) | 891.9 (15.2) | 869.7 (16.0) | 943.1 (35.1) | 898.0 (15.8) |
| 60-89 | 775.3 (19.3) | 869.9 (27.5) | 804.6 (17.0) | 836.5 (17.7) | 929.7 (33.4) | 858.7 (15.4) |
| 30-59 | 718.8 (21.7) | 785.8 (57.8) | 744.0 (26.9) | 730.5 (28.4) | 790.0 (38.2) | 746.0 (23.7) |
| <30 | 630.0 (68.8) | 758.0 (59.3) | 668.2 (52.5) | 664.1 (38.6) | 703.0 (75.9) | 672.8 (37.2) |

Abbreviations: eGFR, estimated glomerular filtration rate; HbA1c, hemoglobin A1c.

**Supplementary Table 2** Mean s-Klotho levels according to renal function in 10,776 NHANES participants without type 2 diabetes.

|  | **eGFR category (ml/min/1.73 m^2^)** | | | | |  |
| --- | --- | --- | --- | --- | --- | --- |
|  | **>120** | **90-119** | **60-89** | **30-59** | **<30** | **p-trend** |
| Mean s-klotho | 953.2 | 868.5 | 834.5 | 758.8 | 617.6 | <0.001 |
| SE | (31.4) | (6.6) | (6.2) | (10.3) | (29.8) |  |

Abbreviations: SE, Standard Error.
